# Association between work status and depression in informal caregivers: a collaborative modelling approach

**DOI:** 10.1093/eurpub/ckab178

**Published:** 2021-11-25

**Authors:** Aoife O’Neill, Stephen Gallagher, Ailish Hannigan, Katie Robinson

**Affiliations:** 1 Ageing Research Centre, School of Allied Health, University of Limerick, Limerick, Ireland; 2 Health Research Institute, University of Limerick, Limerick, Ireland; 3 Department of Psychology, University of Limerick, Limerick, Ireland; 4 Public and Patient Involvement Research Unit, Graduate Entry Medical School, University of Limerick, Limerick, Ireland

## Abstract

**Background:**

Care is regularly provided on an informal basis by family and friends and it is well established that caregivers experience high rates of depression. The majority of research on caregivers tends to focus on older, full-time caregivers, with less attention paid to working caregivers (in paid employment). The aim of this study is to explore the impact of work status on depression in caregivers.

**Methods:**

A sample of individuals from the 2014 European Social Survey dataset, aged 18 and older, who reported being a caregiver, were investigated (*n* = 11 177). Differences in sociodemographic, mental and physical health and social network variables, between working and non-working caregivers, were investigated. Hierarchical logistic regression models were used to investigate associations between the caregivers’ work status and depression. This study was developed in partnership with a panel of caregivers who contributed to the conceptualization and interpretation of the statistical analysis.

**Results:**

Findings showed that 51% of caregivers reported being in paid employment. Non-working caregivers were more likely to be female, older, widowed, have lower education levels and provide intensive caring hours. They were also more likely to report depressive symptoms than working caregivers after controlling for sociodemographic, social networks and intensity of caring (adjusted odds ratio = 1.77, 95% confidence interval = 1.54–2.03). The panel considered policies to support continued work important as a means of maintaining positive mental health for caregivers.

**Conclusions:**

Supportive policies, such as flexible working and care leave, are recommended to allow caregivers to continue in paid work and better manage their health, caring and working responsibilities.

## Introduction

Informal care is regularly provided by family and friends^1^ and plays an essential role in the healthcare system. Informal caring responsibilities often fall disproportionally on certain demographic groups, such as middle-aged women^1^ with lower levels of education.^2^ European differences are evident from a recent publication, reporting that caregivers were most likely to be unemployed women, aged 50–59 years, using European Social Survey (ESS) data.^2^

Links with reduced wellbeing have been identified among caregivers.^2–4^ Informal caregivers, who provide care to a sick or disabled relative, are at an increased risk of depression compared with non-caregivers.^3^ Rates of depression vary within the caregivers’ population, from 29% up to 42%,^5^^,^^6^ which is considerably higher than the prevalence in the general population at 4.4%.^7^ This is a cause for concern as depression in informal caregivers can have negative consequences on both the caregivers’ and the care-recipients’ health and wellbeing.^8^^,^^9^

Sociodemographic factors associated with increased odds of depression in general^10^ and caregiver populations^6^^,^^11^ include lower education, female gender, economic inactivity and being divorced or widowed. Caregivers face further unique caregiving-related risk factors. Increased caregiving stressors, such as physical health symptoms^8^ and caregiver burden,^12^ are associated with depression.

Working caregivers may be at an increased risk as they face the challenge of balancing caring responsibilities with work and other responsibilities. While the strain of this dual role has been discussed,^4^^,^^13^ other studies suggest that caregivers may benefit from paid employment.^14^^,^^15^ Positive links between employment and caregiver wellbeing were identified in a study of parental caregivers of children with intellectual disabilities.^16^ Research found that full-time working caregivers had lower levels of depression, measured on Beck’s Depression Inventory scale compared with caregivers working less than part-time.^16^ Elsewhere, employment was shown to reduce caregivers’ distress.^17^ The benefits of paid employment may include the opportunity to have a role outside of caring, access to workplace-based social support and greater social networks and enhanced economic resources.^15^

A conceptual framework of the challenges faced by those combining work and unpaid care identified multiple interacting challenges including high and/or competing caregiver demands, psychosocial or emotional stressors, the distance between the workplace and care-recipient’s residence and caregiver’s health and financial pressure.^4^ Potential solutions to these challenges include informal or formal help with caring, domestic support, technology, work accommodations, flexible work hours, self-employment and emotional support.^4^

This study was conceptualized and developed in partnership with caregivers, acknowledging and valuing their knowledge in deciding what factors impact their health. While this type of public and patient involvement (PPI) is rare in statistical modelling, it can support collective learning, advance understanding and increase impact.^18^ The combined aims, of the researchers and panel, focus on the health implications for working family caregivers, which builds on previous international research.^19^ The researchers and panel identified two key questions of interest: ‘how do working and non-working caregivers’ differ’? and ‘what is the impact of work status on caregiver’s depression’?

Synthesizing panel feedback with the reviewed literature,^4^^,^^8^^,^^10^ the researchers developed the following refined aims: (i) to identify sociodemographic, mental and physical health and social network differences, between working and non-working caregivers, and (ii) to investigate the impact of work status on caregivers’ depression, using hierarchical logistic regression models and controlling for sociodemographic variables.

## Methods

### Study

This study uses data from the 2014 seventh round of the ESS, which focuses on ‘social inequalities in health and their determinants’. Anonymized data from the ESS are freely available without restrictions for not for profit purposes. The ESS is a biennial cross-national survey of attitudes and behaviour established in 2001. The ESS uses cross-sectional, probability samples, which are representative of all persons aged 15+, resident within private households in each country. ESS is a pan-European survey of 21 countries; Austria, Belgium, Czech Republic, Denmark, Estonia, Finland, France, Germany, Hungry, Ireland, Israel, Lithuania, The Netherlands, Norway, Poland, Portugal, Slovenia, Spain, Sweden, Switzerland and the UK. National co-ordinators and survey agencies ensure compliance with ethics approval procedures at a country level, overseen by the ESS European Research Infrastructure Consortium which subscribes to the Declaration on Professional Ethics of the International Statistical Institute.

Data were collected via face‐to‐face interviews with individuals aged 15+ living in private households. The average response rate for all countries was 51.6%. Data from a total of 35 063 participants were collected. The 2014 ESS was analyzed, as it is the latest round to include data relating to informal caregivers. Complete information on the survey, including questionnaires, is available from the following http://www.europeansocialsurvey.org.

### Sample

A sub-sample of participants from the ESS dataset, aged 18+, who reported being a caregiver, were investigated (*n* = 11 177, 32% of all participants). A caregiver was defined as someone who reported looking after or helping family members, friends, neighbours or others. A non-intensive caregiver was defined as anyone who provided up to 10 h of help a week, while an intensive caregiver was someone who provided over 10 h of help a week. Differences between caregivers and non-caregivers are detailed elsewhere.^2^

### Measures

#### Demographic information 

Demographic information included sex (male, female), age (<25, 25–34, 35–44, 45–54, 55–64, 65–74, 75+ years) and relationship status (married/partner, separated/divorced, widowed, single). The country was categorized into four regional groups^20^: ‘North’ (Denmark, Finland, Norway and Sweden), ‘West’ (Austria, Belgium, France, Germany, Ireland, The Netherlands, Switzerland and UK), ‘Central/East’ (Czech Republic, Estonia, Hungry, Lithuania, Poland and Slovenia) and ‘South’ (Israel, Portugal and Spain). Education was classified using the 2011 International Standard Classification of Education (ISCED). Educational status was categorized as low secondary or less (ISCED I and II), upper secondary (ISCED IIIa, IIIb and IV) and tertiary (ISCED V).^21^

#### Mental health

Depression was assessed using an eight-item version of the Center for Epidemiological Studies Depression Scale.^22^ Individuals were asked how often they felt each of the following in the past week: felt depressed, felt everything was an effort, sleep was restless, was happy, felt lonely, enjoyed life, felt sad and could not get going. Those scoring a value of 10 or more were classified as having depressive symptoms.^20^ The validity and reliability of this scale for depression were previously demonstrated.^23^

#### Physical health

Participants were asked which of the following health problems they have had or experienced in the last 12 months (yes, no), from a list of the following: heart or circulation problem, high blood pressure, breathing problems, back or neck pain, muscular or joint pain in hand or arm, or muscular or joint pain in foot or leg, stomach or digestion related, skin condition related, severe headaches, diabetes and cancer. These conditions were chosen based on prevalence across Europe and common cause of deaths.^24^

#### Social network

Participants were asked how often they socially meet with friends, relatives or colleagues (once a month or less, several times a month, once a week, several times a week/everyday).

#### Work status

Work status was defined where a participant reported their main activity in the last 7 days as paid employment.

### Statistical analysis

The dataset for analysis was pooled across all countries and both post-stratification and population weights were applied to ensure that the survey data represent the national populations of 15+ years with respect to age, gender, education and region and give all countries a weight proportional to population size. Categorical data were described using counts and percentages. Pearson’s *χ*^2^ test was used to test associations between categorical variables. Cramer’s V effect size, with V = 0.1, 0.3 and 0.5 for a small, medium and large effect, respectively, was reported where appropriate. Hierarchical logistic regression models were used to analyze associations between the caregivers’ work status and depression (Model 1), controlling for sociodemographic variables (Model 2) and controlling for intensity of caring and social networks (Model 3). Adjusted odds ratios (AORs), corresponding 95% confidence intervals (CIs) and the Nagelkerke *R*^2^ goodness of fit statistic are reported. A 5% level of significance was used. All statistical analysis was undertaken using SPSS Version 24.

### Public and patient involvement

Two stakeholder panel meetings were held with four caregivers, prior to and after statistical analysis. The panel included four full-time family caregivers; three females and one male, and all were older adults. None of the caregivers were active in the labour market at the time of this study, but three had previously balanced work and care responsibilities. The four caregivers were recruited from a larger PPI panel of older adults who have committed to working with academics on various research projects; panel recruitment is described elsewhere.^25^

The initial meeting focused on discussing experiences of providing care to family members, defining a caregiver and balancing paid work and caregiving. Potential health and demographic differences, between working and non-working caregivers, were considered and factors which influence these differences were identified and discussed, informed by existing literature. This collaborative or participatory modelling approach involves all stakeholders in the model building process, where participants can suggest characteristics for inclusion in the model and how they may impact on the outcome.^26^ Thus, the final variable selection was based on previous research findings, available data, as well as panel feedback.

The second meeting focused on interpreting the results of the statistical analysis and framing discussion points. The PPI meetings were unstructured and facilitated through online video calls. Face-to-face meetings were not possible due to Irish public health restrictions as a result of the COVID-19 pandemic in late 2020. 

## Results

### Sociodemographics

Table 1 presents demographic information on caregivers (*n* = 11 177). The majority are female, middle aged, employed, with an upper secondary education and reported being non-intensive caregivers (77.8%), compared with 21.9% reporting intensive caring over 10 h.

**Table 1:** Demographic information for caregivers (*n* = 11 177) by work status

		**Full sample** **(*n* = 11 177)**	Work status		***P*-values** **(effect size)**
			**Yes** **(*n* = 5743)**	**No** **(*n* = 5409)**	
Sex	Male	5009 (44.8)	2891 (50.3)	2108 (39.0)	<0.001 (0.11)
	Female	6168 (55.2)	2852 (49.7)	3300 (61.0)	
Age	<25	1135 (10.2)	415 (5.8)	719 (12.3)	<0.001 (0.52)
	25–34	1413 (12.6)	988 (18.6)	425 (8.9)	
	35–44	1688 (15.1)	1210 (21.1)	474 (8.8)	
	45–54	2432 (21.8)	1826 (31.8)	596 (11.0)	
	55–64	2254 (20.2)	1175 (20.5)	1072 (19.8)	
	65–74	1471 (13.2)	113 (2.0)	1356 (25.1)	
	75+	785 (7.0)	16 (0.2)	766 (14.1)	
Relationship status	Married/partner	6685 (60.3)	3522 (617)	3146 (58.7)	<0.001 (0.15)
	Single	2793 (25.0)	1508 (9.9)	1279 (9.0)	
	Separated/divorced	1050 (9.5)	566 (1.9)	483 (8.5)	
	Widowed	563 (5.1)	109 (26.4)	454 (23.9)	
Education	Lower secondary or less	3201 (28.8)	1057 (18.5)	2143 (40.0)	<0.001 (0.25)
	Upper secondary	5742 (51.7)	3228 (56.4)	2498 (46.6)	
	Tertiary	2161 (19.5)	1440 (25.2)	714 (13.3)	
Region	North	819 (7.3)	456 (7.9)	363 (6.7)	<0.001 (0.04)
	West	7138 (63.9)	3662 (63.8)	3461 (64.0)	
	Central/east	1715 (15.3)	919 (16.0)	793 (14.7)	
	South	1505 (13.5)	707 (12.3)	792 (14.6)	
Hours spent caring	Non-intensive caregiver (1–10** **h)	8594 (77.8)	4743 (83.5)	3833 (71.8)	<0.001 (0.14)
	Intensive caregivers (>10** **h)	2451 (22.2)	937 (16.5)	1507 (28.2)	

Counts (%) presented.

Sociodemographic differences between working and non-working caregivers are evident from table 1. Working caregivers are more likely to be male, middle aged, have a higher level of education and provide non-intensive caring hours. Non-working caregivers are more likely to be female, older, widowed, have lower education levels and are more likely to provide intensive caring hours.

### Physical health, depression status and social networks

Table 2 presents caregivers with a physical or mental health complaint, by work status. For all caregivers, 12.9% report symptoms of depression, with a statistically significant difference between working and non-working caregivers (16.5% of non-working caregivers reporting depression compared with 9.6%, *P* < 0.001).

**Table 2:** Caregivers reporting a mental or physical health condition, by work status

	**Full sample** **(*n* = 11 177)**	Work status		***P*-values** **(effect size)**
		**Yes** **(*n* = 5743)**	**No** **(*n* = 5409)**	
Mental health				
Depression	1426 (12.9)	546 (9.6)	878 (16.5)	<0.001 (0.10)
Physical health				
Heart or circulation problem	1243 (11.2)	367 (6.4)	876 (16.2)	<0.001 (0.16)
High blood pressure	2117 (19.0)	742 (13.0)	1372 (25.4)	<0.001 (0.16)
Breathing problems	1191 (10.7)	512 (8.9)	674 (12.5)	<0.001 (0.06)
Back or neck pain	5355 (48.1)	2840 (49.6)	2502 (46.4)	0.001 (0.03)
Muscular or joint pain in hand/arm	3094 (27.8)	1430 (25.0)	1657 (30.7)	<0.001 (0.06)
Muscular or joint pain in foot/leg	3088 (27.6)	1384 (24.2)	1697 (31.4)	<0.001 (0.08)
Stomach or digestion related	2230 (20.0)	1056 (18.5)	1174 (21.8)	<0.001 (0.04)
Skin condition	1209 (10.9)	546 (9.5)	658 (12.2)	<0.001 (0.04)
Severe headache	2074 (18.6)	1075 (18.8)	999 (18.5)	0.72 (0.003)
Diabetes	648 (5.8)	187 (3.4)	450 (8.3)	<0.001 (0.11)
Cancer (currently)	390 (3.6)	130 (2.3)	259 (4.9)	<0.001 (0.07)

Count (%) presented.

Back or neck pain is the most prevalent health complaint (48.1%). Across nearly every health complaint there is a statistically significant difference between prevalence for working and non-working caregivers with those not working more likely to report the health complaint.

Table 3 presents caregivers’ social networks, by work status. Non-working caregivers are most likely to report frequent social meetings, with 48.1% reporting socializing several times a week/everyday compared with 41.8% of working caregivers.

**Table 3:** Caregivers’ social network, by work status

		**Full sample** **(*n* = 11 177)**	Work status		***P*-values** **(effect size)**
			**Yes** **(*n* = 5743)**	**No** **(*n* = 5409)**	
Social meetings	Once a month or less	1827 (16.4)	935 (16.4)	884 (16.4)	<0.001 (0.07)
	Several times a month	2281 (20.5)	1236 (21.6)	1042 (19.4)	
	Once a week	2019 (18.2)	1154 (20.2)	864 (16.1)	
	Several times a week/everyday	4985 (44.9)	2387 (41.8)	2586 (48.1)	

Count (%) presented.

### Hierarchical logistic regression model of depression

Table 4 presents the hierarchical logistic model predicting depression for caregivers. Model 1 suggests non-working caregivers are at an increased risk of being depressed, when compared with working caregivers (OR = 1.92, 95% CI = 1.71–2.15). This increased risk is consistent across the hierarchical models, when controlling for demographic variables (Model 2; AOR = 1.82, 95% CI = 1.59–2.09) and intensity of caring and social networks (Model 3; AOR = 1.77, 95% CI = 1.54–2.03). Country differences were identified with an increased risk of depression of non-working carers in countries in all regions compared with the North region (Denmark, Finland, Norway and Sweden).

**Table 4:** Hierarchical logistic regression model of depression

		Model 1		Model 2		Model 3	
		OR (95% CI)	*P*-values	OR (95% CI)	*P*-values	OR (95% CI)	*P*-values
Non-working caregiver		1.92 (1.71–2.15)	<0.001	1.82 (1.59–2.09)	<0.001	1.77 (1.54–2.03)	<0.001
Female				2.00 (1.76–2.27)	<0.001	1.99 (1.75–2.26)	<0.001
Age (ref. <25)	25–34			1.53 (1.18–1.98)	<0.001	1.50 (1.16–1.94)	<0.001
	35–44			1.85 (1.41–2.43)		1.82 (1.38–2.39)	
	45–54			1.51 (1.15–1.98)		1.47 (1.12–1.93)	
	55–64			1.26 (0.96–1.66)		1.22 (0.29–1.61)	
	65–74			0.86 (0.63–1.16)		0.83 (0.62–1.13)	
	75+			1.05 (0.76–1.47)		1.03 (0.74–1.44)	
Education (ref. tertiary)	Primary			3.23 (2.63–3.98)	<0.001	3.07 (2.49–3.78)	<0.001
	Secondary			2.00 (1.64–2.45)		1.97 (1.61–2.41)	
Relationship status (ref. married/partner)	Separated/divorced			1.73 (1.43–2.09)	<0.001	1.73 (1.43–2.09)	<0.001
	Widowed			1.62 (1.34–2.08)		1.63 (1.33–2.09)	
	Single			1.59 (1.34–1.89)		1.59 (1.33–1.89)	
Region (ref. North)	West			2.40 (1.75–3.29)	<0.001	2.37 (1.73–3.26)	<0.001
	Central/East			2.72 (1.94–3.83)		2.53 (1.79–3.58)	
	South			2.58 (1.83–3.64)		2.42 (1.71–3.42)	
Intensive caregiver						1.27 (1.11–1.45)	<0.001
Social meetings (ref. several times a week/everyday)	Once a month or less					1.23 (1.04–1.45)	<0.001
	Several times a month					0.65 (0.55–0.77)	
	Once a week					0.89 (0.75–1.05)	
Nagelkerke *R*^2^	0.02			0.09		0.10	

Model 2 controlling for sex, age, relationship status, education and region.

Model 3 controlling for sex, age, relationship status, education, region, intensity of caregiving and social meetings.

## Discussion

This study examined data from the 2014 ESS and focused on the health implications for working and non-working caregivers. Within ESS, 11 177 (32%) were characterized as informal caregivers, with over half (51%) also being in paid employment. Results found that non-working caregivers are at a considerably higher risk of depression compared with working caregivers (16.5% of non-working caregivers reported depressive symptoms compared with 10% of working caregivers). After controlling for sociodemographic variables, intensity of caring and social networks, non-working caregivers were more likely to report depressive symptoms than working caregivers (AOR = 1.77, 95% CI = 1.54–2.03).

This study was conceptualized and interpreted with a panel of caregivers. The finding of non-working caregivers being more at risk of depression resonated with the panel, but they considered the prevalence of depression somewhat lower than expected. While higher rates of depression were reported in reviews of caring populations,^5^^,^^6^ these reviews included few studies with population representative samples. Similar rates of depression to our findings were reported in other studies using population-representative samples.^27^^,^^28^

The panel highlighted that working caregivers have the ability to physically and mentally leave their caring responsibilities temporarily, they feel more independent and important, they have opportunities to make money, get dressed up and have social interactions at work. They discussed the mental health impact of being a full-time caregiver: feeling isolated, lonely, invisible, guilty and misunderstood; feelings which are commonly reported in other qualitative studies of family caregivers.^29–31^ The panel detailed the benefit of work’s social experience for their mental health. This is in accordance with our statistical findings showing links between social networks and depression. Elsewhere in the literature, results suggest that long-term activity restrictions are related to increased depression in caregivers.^8^

Despite the benefits of working, our panel highlighted that, depending on the amount of caring being provided, balancing work and caring responsibilities is not feasible long term. They reported feeling like they ‘lived two separate lives’, one at work and one at home. This was a cause of stress and anxiety, as they felt conflicted about going to work as they were needed at home. Generally, the PPI panel believe the support structures are not in place to support working caregivers and reported feeling under-valued by the government.

The panel made recommendations towards flexible working conditions such as the option to work from home and also acknowledged the positive effects support groups and good social networks have on caregivers’ mental health. This aligns with previous research, which reported the protective impact of social networks for caregivers’ mental health^32^ and our findings that caregivers who had more social contact were less depressed. Yet, while the panel considered work as an important means of facilitating impromptu social interactions, our statistical results suggested that non-working caregivers had more social meetings. Public health campaigns or interventions focusing on improving social networks for caregivers have previously been recommended^32^ and may contribute to raising awareness of the importance of strong social networks for caregiver’s mental health.

Verbakel et al.^2^ made general recommendations towards supportive policies, such as respite care, training or counselling, being made more easily accessible to caregivers. However, we must consider how current supportive policies for caregivers vary considerably across Europe.^33^^,^^34^ While financial support is the most common type of support provided,^33^ findings suggest that more effective supports are those that give a break from caring responsibilities, support caregivers emotionally and provide them with skills to improve and better deal with their care situation.^35^ Our findings suggest working caregivers have better mental health. Thus implementing more standardized policies could aid working caregivers in balancing their dual responsibilities and better sustain informal care, which is an important resource for our healthcare systems.

Since data collection in 2014, policy changes have been primarily at individual country level with heterogeneous policies in place. However, on 20 June 2019, the European Union Directive on work–life balance for parents and carers introduced the entitlement to 5 days of carers’ leave per year, for workers providing personal care or support to a relative or person living in the same household and extended the right to request flexible working arrangements to working carers.^36^ While many EU Member States already have measures in place that go beyond these provisions, the Work–Life Balance Directive can nevertheless be considered an important step in recognizing informal carers.

Recently, Brimblecombe et al.^37^ discussed the idea of reducing the need for unpaid care. They recognized the substantial costs incurred by governments for caregivers, through lower tax revenue, welfare benefit payments and health service use. While these costs are essential to support caregivers, the question was raised as to whether these funds could be better spent on supportive policies. Initiatives could be developed to support the education, training, employment, financial situation and physical and mental health of caregivers. More specifically Brimblecombe et al. highlighted how support in workplaces is valuable to working caregivers, a point consistent with our PPI panel. Here, the panel considered flexible working essential, as they believe the cost of a replacement or substitute caregiver during work hours does not equate to the income earned. The panel noted the ability to work at home in some capacity was more suited to facilitating working and caring responsibilities.

A European report strengthens this claim by suggesting suitable interventions, to facilitate caregivers combining work and care including care leave and making work flexibility legally possible.^34^ While it is clear that work support is fundamental to caregivers’ wellbeing, evidence also suggests a combination of potentially effective interventions is most effective.^38^ Other suitable support policies for working caregivers’ wellbeing could include combinations of formal care services for people with care needs (‘replacement’ or ‘substitution’ care), psychological therapy, training and education, and support groups.

The COVID-19 pandemic has accelerated changes to the ways people work and these changes have the potential to create additional challenges and/or potential benefits for working caregivers.^39^ Further research is needed on the longitudinal impact and differential impact of the pandemic on working family caregivers.^39^

### Strengths and limitations

A unique strength of this study is the collaboration between a PPI panel of family caregivers and academic researchers. PPI in the statistical analysis is often underexplored but acknowledging and valuing lay knowledge of the context supported meaningful interpretations of our findings. Another strength is the use of data from a large pan-European study of 21 countries, providing useful insights into the health implications for working and non-working caregivers.

Rates of depression are somewhat lower than previously identified in caregiver populations.^5^^,^^6^ Thus, our findings may be somewhat conservative due to the self-reported nature of the ESS data and the lack of data collected on specific caring responsibilities. For example, the caring role and the hours provided are both self-reported, meaning some undefined caregivers may be excluded from analysis and information on who is being cared for (e.g. adults and/or children; live-in care vs. care outside the home) is not reported and therefore cannot be accounted for in the analysis.

The panel hypothesized differences in stress by work status; however, no measure of stress was collected in the ESS and therefore could not be incorporated into the analysis. Future research could consider working collaboratively with a panel of caregivers, prior to data collection to expand on the variables to be measured and increase explanatory ability of the statistical models. The cross-sectional nature of the data is also a limitation, as no conclusions can be made as to the long-term impact of caring on mental health. The data collection date (2014) means that changes in social policy to support carer participation in the labour market in Europe^40^ in the intervening years would not be reflected in the statistical findings. While several significant associations of depression are identified, a substantial proportion of the variance in the models is unexplained.

While none of the four caregivers involved in the panel was working at the time of the study, three had previously balanced work and caring commitments. As circumstances, attitudes and legislation may change over time, the perspective of caregivers active in the labour market may have resulted in alternative feedback. Due to the timing of this study, with COVID-19 restrictions in place, it was not feasible to recruit additional caregivers to the already established research panel.^25^ We would recommend future work consider a similar collaborative modelling approach with a mix of caregivers who are both active and inactive in the labour market. The insights provided by the caregiver panel may be restricted to an Irish focus. However, legislative and normative contexts with regard to labour market participation, care provision may differ across the other ESS represented countries in Europe. We identified cross-country differences in our study and further subgroup analysis of region may be useful to identify any effect modification for caregivers’ depression with a more detailed analysis of cross-country differences in caregiver legislation and consulting caregivers from across Europe.

## Conclusions

In a study of 11 177 caregivers, from the 2014 ESS, differences between working and non-working caregivers were evident. The findings were interpreted in partnership with a panel of caregivers, highlighting the value of collaborative modelling. Findings suggest that non-working caregivers are at a considerably higher risk of depression when compared with working caregivers. Supportive policies such as flexible working and care leave are recommended. Enabling caregivers to continue in paid work and better balance their caring and working responsibilities would support caregivers’ health and sustain an important resource for our healthcare systems.
